# Aromatic disulfides as potential inhibitors against interaction between deaminase APOBEC3G and HIV infectivity factor: Inhibitors of interaction between APOBEC3G and Vif

**DOI:** 10.3724/abbs.2022049

**Published:** 2022-05-25

**Authors:** Xiaoxuan Yan, Chao Chen, Chunxi Wang, Wenxian Lan, Jianguo Wang, Chunyang Cao

**Affiliations:** 1 State Key Laboratory of Bioorganic and Natural Products Chemistry Shanghai Institute of Organic Chemistry Center for Excellence in Molecular Synthesis Shanghai Institute of Organic Chemistry Chinese Academy of Sciences Shanghai 200032 China; 2 State Key Laboratory of Elemento-organic Chemistry National Pesticide Engineering Research Center College of Chemistry Nankai University Tianjin 300071 China; 3 University of Chinese Academy of Sciences Beijing 100049 China

**Keywords:** APOBEC3G, Vif, interaction, HIV-1, aromatic disulfide

## Abstract

APOBEC3G (A3G) is a member of cytosine deaminase family with a variety of innate immune functions. It displays activities against retrovirus and retrotransposon by inhibition of virus infectivity factor (Vif)-deficient HIV-1 replication. The interaction between A3G N-terminal domain and Vif directs the cellular Cullin 5 E3-ubiquitin ligase complex to ubiquitinate A3G, and leads to A3G proteasomal degradation, which is a potential target for anti-HIV drug. Currently, there are very few reports about stable small molecules targeting the interaction between A3G and Vif. In this study, we screened two series of small molecules containing carbamyl sulfamide bond or disulfide bond as bridges of two different aromatic rings. Five asymmetrical disulfides were successfully identified against interaction between A3G and Vif with the IC _50_ values close to or smaller than 1 μM, especially, not through covalently binding with A3G or Vif. They restore the A3G expression in the presence of Vif by inhibiting Vif-induced A3G ubiquitination and degradation. This study opens a way to the discovery of new anti-HIV drugs.

## Introduction

APOBEC3G (A3G) catalyzes the deamination of cytosines in HIV-1 gene DNA, and effectively inhibits the replication process of HIV-1 virus. HIV-1 suppresses the inhibitory effect of A3G through its virus infectivity factor (Vif) protein. Vif is involved in the intracellular proteasome degradation pathway to degrade A3G in host cells, which promotes the virus replication successfully. Vif interacts with A3G and recruits E3 ubiquitin ligase complexes. These complexes include cullin5 scaffolding proteins (CUL5), the substrate adaptation extension factor B (elongin B, ELOB) and extension factor C (elongin C, ELOC). In addition, the core-binding factor subunit β (CBF-β) expressed by the host cells is also recruited to form the ubiquitin ligase complex, which promotes the polyubiquitination and degradation of A3G and destroys A3G-mediated cellular defense functions [1]. *In vitro* experiments show that HIV-1 virions containing Vif significantly reduce the cytosine deamination activity of A3G [ 2, 3]. In Vif-contained HIV-1 infected cells, Vif reduces the frequency of G→A mutation induced by A3G in the genome, even if A3G gene is packaged into the virion [4]. In addition, clinical and experimental data demonstrate that virions containing A3G still have the infection ability [ 4, 5], which suggests that Vif can antagonize A3G in other ways, such as inhibiting the translation of *A3G* mRNA, blocking A3G from participating in the capsidation process of virions, and weakening its deamination activity [6].

The potential anti-HIV-1 activity of A3G inspires researchers to screen and develop small molecules that can enhance A3G activity or antagonize Vif function. For example, in virtue of FRET-based high-through screening (HTS) *in vitro*, chemical compound redoxal was identified as an inhibitor of HIV-1 replication in peripheral blood mononuclear cells through inhibition of pyridine biosynthesis [7]. The camptothecin analog O2-16 was recognized to inhibit HIV-1 infection by blocking Vif aggregation [8]. Small molecule N.41 and its analogues were found to block interaction between A3G and Vif with an IC _50_ value of 2.18 μM [9]. However, these compounds are all composed of aromatic rings connected by the framework of enamine ketone which is instable. Therefore, even though the antiviral activity of this class of inhibitors is promising, their structures are required to be optimized [10]. Based on the fluorescent intensity of 293T cells, Vif inhibitor RN-18 was reported to inhibit Vif activity with an IC _50_ value of 4.5 μM [11], which has three aromatic rings, *i*. *e*., A, B and C rings. The A/B rings and B/C rings are connected by sulfur ether bond and amide bond, respectively. By modification of bridge bond of A/B and B/C rings, soluble RN-18 derivatives RN-17 and RN-19 do not display a decreased IC _50_ value [12], while RN-13A has an EC _50_ value equal to 0.25 μM. Further studies suggested that these derivatives did not block the interaction between Vif and A3G, but interrupt the interface of Vif-ELOC [13]. Similarly, compounds IBM-26 and IBM-35 containing two aromatic rings connected by amide or ester bond were found to directly interact with A3G [14]. Using GFP-fused A3G (A3G-GFP) to monitor A3G expression, small molecules MM-1 and MM-2 with benzimidazole skeleton impaired HIV-1 activity by increasing A3G expression rather than interrupting interaction between A3G and Vif [15]. Benzimidazole ring-contained compound ZBMA-1 was reported to bind to the residue Asp ^111^ in ELOC to prevent interaction between Vif and ELOC [16]. However, further structural and relational (SAR) studies of ZBMA-1 demonstrated that small molecules benzimidazole-14 and benzimidazole-26 could disrupt the interaction between A3G and Vif [17]. Dependent on the virtual screening assay, chemicals VEC-5 and Zif-15 with two or three aromatic rings were found to inhibit Vif activity by targeting the interaction between Vif and ELOC [ 18, 19]. Natural product Hop-8 with a long aliphatic chain was found to exert anti-HIV-1 and HIV-2 activities by up-regulating *A3G* mRNA expression [20]. Two natural products Baculiferin L and M, pyrrole alkaloids, isolated from the Chinese marine sponge Iotrochota baculifera, exhibit high inhibition activities countering HIV-1 replication in MT4 and MAGI cells with an IC _50_ value of 0.5–7 μg/mL, by strong interactions with both A3G and Vif [21]. Chemicals TPEN and SN-2 were identified to liberate antiviral function of A3G by chelation with zinc ion in Vif and further disrupting its zinc finger structure [ 22, 23]. The high HIV-1 mutation rates also contribute to the ability of the virus to escape from immune responses and evolve resistance to antiretroviral drugs. Different from the efforts mentioned above, Harris *et al*. reported that the chemical MN30 [24] and its derivates MN132.0262 [25] and MN256.0102 [26] could inhibit A3G activity by covalently binding to its residue Cys ^321^.

Among all these reported small molecule inhibitors, it is not uncommon to have multiple aromatic rings bridged by disulfide or ester bonds as their skeleton. Due to their reported anti-virus activities [ 27– 29], in this report, we screened two series of these small molecules, which included two different aromatic rings connected by ammonia formyl sulfanilamide bond or disulfide bond. Five chemicals were finally found to inhibit A3G degradation by disrupting A3G-Vif interaction.

## Materials and Methods

### Expression and purification of A3G-sNTD

The sequence encoding A3G-sNTD protein was cloned into pGEX-6P-1 vector expressing GST-A3G fusion protein, which contains a PreScission cleavage site in the connection sequence. Then, the plasmid was transformed into *Escherichia coli* BL21(DE3) cells and grew in LB media at 37°C until the OD _600_ reached 0.6. The culture temperature was decreased to 16°C and the cells were induced with 0.2 mM IPTG for 20 h. The collected cell pellets were resuspended in buffer A (50 mM Na _2_HPO _4_, pH 7.3, 150 mM NaCl, 10 μM ZnCl _2_, 0.005% Tween-20, and 1 mM DTT) and lysed at 15 kpsi using a hydraulic cell disruption system (Constant System JINBO Benchtop; Guangzhou Juneng Biology and Technology Co., Ltd, Guangzhou, China). After centrifugation, the supernatant of the cell lysates was incubated with glutathione resin (GE Healthcare, Bethesda, USA), and washed with 5–10 column volumes of buffer A, then digested overnight by PreScission protease in buffer B (50 mM Na _2_HPO _4_, pH 7.3, 100 mM NaCl, 10 μM ZnCl _2_, 0.005% Tween-20, and 1 mM DTT). The elution of the cleaved A3G-sNTD protein was further purified by Superdex75 16/300 GL column (GE Health). Purified protein was analyzed by running SDS-PAGE gel which was stained with Coomassie blue.

### Expression and purification of Full-length APOBEC3G

The sequence encoding full-length APOBEC3G (A3G-FL) with a C-terminal 6× His tag was cloned into the Baculovirus vector pFastBac using the Bac-to-Bac system. The constructed Bacmid DNA was transfected into Sf9 cells by Cellinfectin ( Thermo Fisher Scientific, Waltham, USA) at the cell density of 1.0×10 ^7^ cells/mL. Sf9 cells were cultured at 27°C for 72–96 h at 260 rpm. The cell suspensions were centrifuged and the viruses in the supernatant were collected to further infect Sf9 cells. Then, Sf9 cells were cultured at 27°C for 72 h at 130 rpm. Cells were then collected by centrifugation and resuspended in buffer C (25 mM HEPES, pH 7.2, 1 M NaCl, and 10 mM β-mercaptoethanol) containing protease inhibitors cocktail (Sangon Biotech, Shanghai, China). Triton X-100 was added into the cell lysates at the final concentration of 0.4% (v/v). Then 2 mg RNase A and DNase I was added into the lysates. The lysates were then incubated at 37°C for 30 min and diluted with buffer C containing 1 M urea. Cell lysates were centrifuged at 12,000 rpm for 1 h at 4°C to remove cellular debris prior to loading into a Ni-NTA resin column (GE Health). The colmun was washed with buffer C containing a decreasing concentration gradient of urea from 1 M to 250 mM, and then with 5 column volumes of buffer D (25 mM HEPES, pH 7.2, 500 mM NaCl, and 10 mM β-mercaptoethanol). The column was washed with buffer D containing 70 mM imidazole, and then eluted with buffer D containing 100 mM and 300 mM imidazole successively. Fractions containing A3G-FL were further purified on a Superdex200 Increase 10/300 column pre-equilibrated with buffer E (25 mM HEPES, pH 7.2, 300 mM NaCl, 5% glycerol, and 10 mM β-mercaptoethanol). The purified protein was stored in –80°C prior to use.

### Expression and purification of VCBC complex

The cDNAs of full-length Vif (residues 1–192) and human CBF-β (residues 1–170) were sub-cloned into the vectors pET-Duet-1(MCS-1), pET28a, and pGEX6P-1, respectively, and human non-tagged ELOB (residues 1–102) and ELOC (residues 17–112) were sub-cloned into MCS1 (multiple cloning site 1) and MCS2 of pACYCDuet-1 (Novagen, Gibbstown, USA), respectively. The Vif, CBF-β and ELOB-ELOC constructs were co-expressed in *E*. *coli* BL21(DE3) cells. Expression of the recombinant proteins was induced by 0.3 mM isopropyl β-D-1-thiogalactopyranoside (IPTG) at 16°C for 20 h. Then cells were collected by centrifugation, and resuspended in buffer F (25 mM Tris, pH 8.0, 500 mM NaCl, and 3 mM DTT) supplemented with protease inhibitor phenylmethanesulphonylfluoride (PMSF; Sigma, St Louis, USA). The cells were then lyzed at 15 kpsi using a hydraulic cell disruption system (Constant System JINBO Benchtop; Guangzhou Juneng Biology and Technology Co, LTD) and cell debris was removed by centrifugation at 12,000 rpm for 1 h at 4°C. The hetero-tetrameric protein of Vif-CBFβ-ELOB-ELOC (VCBC) was first purified using Ni-NTA resin (Qiagen, Hilden, Germany) which was washed and eluted with buffer E containing 20 mM, 50 mM, 100 mM, 300 mM and 500 mM imidazole successively. Fractions containing VCBC complex were subjected to treatment with 0.1% polyethylenimine (PEI) to remove nucleic acids in the complex until no more production of white precipitations. Then the supernatant was further purified by Superdex75 16/300 GL column (GE Health) pre-equilibrated with buffer G (25 mM Tris, pH 8.0, 150 mM NaCl, and 3 mM DTT.)

### Expression and purification of Vif

The cDNAs of full-length Vif (residues 1–192) was sub-cloned into the vector pET28a and expressed in *E*. *coli*BL21(DE3) at 37°C until the OD _600_ reached 0.6. The recombinant Vif was induced by 0.5 mM IPTG at 37°C for 4 h. The collected cell pellets were resuspended in buffer H (10 mM Tris-HCl, pH 7.4, 6 M guanidine-HCl) and lysed at 15 kpsi using a hydraulic cell disruption system (Constant System JINBO Benchtop; Guangzhou Juneng Biology and Technology Co., Ltd). The cell lysates were centrifuged at 12,000 rpm for 1 h at 4°C after stirring at room temperature overnight. Then the supernatants were incubated with Ni-NTA resin (GE Healthcare) and washed with 10 column volumes of buffer I (100 mM Na _2_HPO _4_, pH 6.9, 10 mM Tris-HCl, and 8 M urea), then Vif was eluted with buffer J (100 mM Na _2_HPO _4_, pH 4.5, 10 mM Tris-HCl, and 8 M urea). Factions containing Vif was renatured by stepwise dialysis against buffer K (10 mM Tris-HCl, pH 6.0, 100 mM Na _2_HPO _4_, 150 mM NaCl, and 20% glycerol) supplemented with 6 M, 4 M, 2 M, 1 M, and 0.5 M urea. The final step of renaturation of Vif was finished by dialysis against buffer L (10 mM Na _2_HPO _4_, pH 6.0, 150 mM NaCl, and 20% glycerol) to obtain renatured Vif (rVif).

### Surface plasmon resonance (SPR) assay

Binding between A3G-sNTD and rVif was analyzed at 25°C using a Biacore T200 surface plasmon resonance biosensor (GE Healthcare). rVif was captured by anti-His antibodies coupled to a CM5 sensor chip at 180 resonance units (RU) at the flow rate of 10 μL/min and contact time of 60 s. Then threefold serial dilutions of freshly prepared A3G-sNTD were injected at a flow rate of 30 μL/min (120 s contact time). PBS-P was used as the running buffer and the sensor chip was regenerated by glycine (pH 1.5). The data analyses were performed by BIAcore Evaluation with kinetic mode to calculate the *K*_D_, *k*_d_ and *k*_a_ values.

Small molecules screening was performed in PBS-P buffer with 5% DMSO. rVif was captured by anti-His antibodies immobilized to an CM5 sensor chip at the flow rate of 10 μL/min and contact time of 60 s. Small molecules in the concentration of 500 μM were injected at 30 μL/min. The contact time is 100 s and dissociation time is 120 s. The chip was regenerated by glycine (pH 1.5) at a flow rate of 30 μL/min (30 s contact time). Then small molecules with high RU were selected to perform the 2 ^nd^ round screening. Two-fold serial dilutions from 100 μM of small molecules were injected at a flow rate of 30 μL/min (60 s contact time). The data analyses were performed by BIAcore Evaluation.

In the competitive binding assay, A3G-FL protein was immobilized to a CM5 sensor chip to a level of ~104 response units (RUs). Two-fold serial dilutions of chemicals were injected together with 32 nM VCBC complex in concentrations ranging from 16 nM to 2516 nM at a flow rate of 30 μL/min. The contact time is 100 s and dissociation time is 120 s. The RUs were plotted against the concentration of small molecules and *K*_i_ was calculated by GraphPad Prism 8.0.

Binding between A3G-FL and VCBC complex was also analyzed at 25°C using a BIAcore T200 surface plasmon resonance biosensor (GE healthcare). A3G-FL coupled to a CM5 sensor chip at 104 resonance units (RU) at the flow rate of 10 μL/min and contact time of 60 s. Two-fold serial dilutions starting from 500 nM of freshly prepared VCBC complex were injected at a flow rate of 30 μL/min (120 s contact time). The A3G-FL sensor chip was regenerated by glycine (pH 1.5). Responses from the protein surface were corrected for the response from the mock surface and for responses from a separated buffer only injection. The data analyses were performed by BIAcore Evaluation with kinetic mode to calculate the *K*_D_, *k*_d_ and *k*_a_ values. The saturating concentration of VCBC complex against A3G-FL was analyzed and selected as the final concentration in the competitive binding SPR assay.

### Cell culture and transfection

HEK 293T cells were cultured in DMEM medium supplemented with 10% FBS (Thermo Fisher Scientific) at 37°C, 5% CO _2._. The plasmids were transfected into cells using Lipo6000 ^TM^ transfection reagent (Thermo Scientific) according to the manufacturer’s instructions.

### Fluorescence intensity-based drug screening assay and quantification

Fluorescence intensity-based drug screening assay was performed as previously reported [24]. The sequences encoding A3G-FL and Vif were cloned into the vectors pEGFP-N1 and pcDNA3.1-HA, respectively. Cells were seeded into 12-well plate at cell density of 0.7×10 ^6^ cells/well, and transfected with pEGFP-N1-A3G and pcDNA3.1-HA-Vif plasmids. After 12-hour transfection, the media were replaced by medium containing 10 μM small molecules and the cells were further incubated for another 24 h.

Cells were assessed visually under a fluorescence microscope, and lysed with RIPA buffer (Beyotime, Nantong, China). After lysis, the fluorescence intensity of samples was measured and analyzed with the Envision Multimode Plate Reader (Perkin-Elmer, Boston, USA). The intensity of cell samples was normalized and average fluorescence intensity of A3G and pcDNA3.1-HA co-transferred cells was set as 100%.

### Immunoprecipitation and western blot analysis

Cells were lysed with RIPA lysis buffer (Beyotime) supplemented with protease inhibitor cocktail (Sangon Biotech). After centrifugation at 12,000 rpm for 20 min, the supernatant was mixed with loading buffer, boiled for 10 min, resolved on 10% (w/v) polyacrylamide gel and transferred to PVDF membrane (Millipore, Billerica, USA). The membrane was blocked with 5% non-fat milk for 1 h and analyzed with standard western blotting procedures. Primary antibodies against EGFP, Vif and β-actin were purchased from Abcam (Cambridge, UK). HRP-conjugated secondary antibodies were purchased from Sangon Biotech.

To test the ubiquitination of A3G, HEK 293T cells were transfected with expression vectors (pcDNA3.1-Ubiquitin, pEGFP-N1-A3G) for HA-tagged ubiquitin and EGFP-fused A3G, with or without co-transfection of the expression vectors (pcDNA3.1-Vif and pcDNA3.1-HA) for Vif, respectively. After 10 h of incubation at 37°C and 5% CO _2_, the media were replaced by medium containing 20 μM small molecules. Cells were treated with 2.5 μM MG132 for 16 h before harvest, then washed with PBS, lysed with RIPA buffer (25 mM HEPES, pH 7.4, 150 mM NaCl, 0.1% SDS, 0.1% sodium deoxycholate, 1% triton X-100, and 1 mM EDTA) supplemented with protease inhibitor cocktail and 10 μM MG132, centrifuged for 20 min at 20,000 *g*. The supernatant was incubated with anti-GFP rabbit polyclonal antibody (Thermo Fisher Scientific) overnight, and then mixed with 50 μL Dynabeads Protein A (Invitrogen, Carlsbad, USA) over night. Beads were washed with PBS (pH 7.4) containing 0.02% Tween-20 for three times. Twenty microliters of PBS (pH 7.4) containing 0.02% Tween-20 was added to resuspend the Dynabeads-Ab-Ag complex, and the prepared samples were analyzed by western blotting.

## Results

### Compounds interact with both rVif and A3G-sNTD *in vitro*

**Figure 1:**
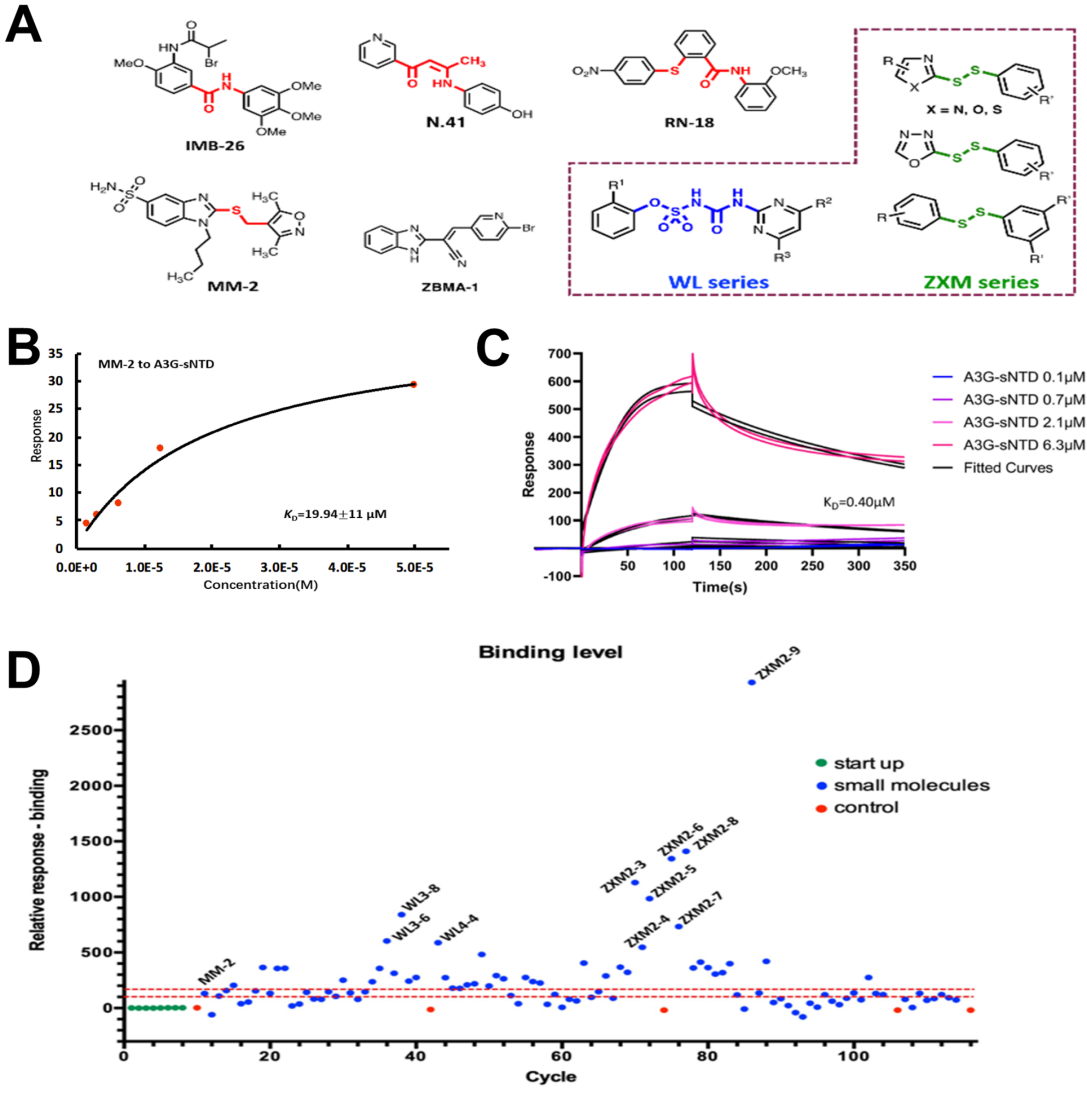
Figure1 Chemicals were found to bind to A3G-sNTD or rVif (A) Chemical library design of WL and ZXM series of compounds (highlighted in dashed box) based on the reported aromatic inhibitors. (B) The binding affinity of MM-2 to A3G-sNTD measured by SPR assay. (C) Kinetics fitting of A3G-sNTD binding with rVif. (D) Response levels of interactions between rVif and different small molecules. Vertical axis indicates the response unit of rVif chips with small molecules flow through; horizontal axis shows the cycle numbers during SPR assay. In (D), different cycles stand for different samples; blue dots represent samples with small molecules; green dots represent starting-up samples at the beginning of experiment; red dots represent samples for correction every 32 cycles; red dashed lines indicate RU=150 and RU=100 respectively. Small molecules with high response are labeled.

The reported compounds IMB-26, N.41, ZBMA-1, MM-2 and RN-18 ( Figure 1A) with two or three aromatic rings are able to inhibit HIV-1 activities. The aromatic rings in these compounds are connected by the chemical bonds between nitrogen or sulfur atoms or vinyl group. MM-2 displays a binding affinity to A3G-sNTD with a *K*_D_^MM-2, A3G-sNTD^ of 19.94±11.00 μM ( Figure 1B). These results suggest that aromatic rings are essential elements for the inhibitors which interact with A3G or Vif. In this study, we constructed a chemical library including 2 series of compounds in our lab, namely WL and ZXM series. Both contain two different aromatic rings connected by ammonia formyl sulfanilamide bond or by disulfide bond. The WL compounds represent 38 sulfonylurea-containing chemicals ( Supplementary Table S1), which were ever reported as novel antifungal agents targeting acetohydroxyacid synthase [27]. The ZXM series include 59 disulfide-bond-containing compounds ( Supplementary Table S2), which were ever reported as novel inhibitors of SARS-CoV main protease [28] or acetohydroxy acid synthase [29].

Since we cannot obtain soluble N-terminal domain of A3G ( *i*. *e*., A3G-NTD), we used A3G-sNTD to replace A3G-NTD. Although A3G-sNTD is a A3G-NTD mutant with 20% residues’ mutations in the N-terminus of wild-type A3G-NTD as previously described [30], these mutations could increase the binding affinity of A3G-NTD to Vif. The recombinant Vif (rVif) was obtained through refolding of inclusion bodies produced from *E*. *coli* system. The binding affinity of Vif to A3G-sNTD was tested by SPR assay. The data indicated that rVif had a strong binding affinity to A3G-sNTD with a *K*_D_^A3G-sNTD, rVif^ of 0.40 μM ( Figure 1C), and with an association constant ( *k*_a_) of 92 M ^-1^s ^-1^ and a dissociation constant ( *k*_d_) of 0.0037 s ^-1^. These results indicate that A3G binds quickly to rVif, but slowly dissociates from rVif.

Some chemicals were then screened out from WL and ZXM series, which possibly interact with rVif using SPR with a positive control molecule, MM-2. The relative response value of MM-2 binding to rVif is 130.4 resonance units (RU) ( Figure 1D and Supplementary Tables S1 and S2). We found that most of WL and ZXM compounds had response values between 100 and 150 RU. In theory, the compounds may have no interactions with rVif when their response values are smaller than 100 RU, while the chemicals may have moderate interactions with rVif when their response values are between 100 and 150 RU. Definitely, if the response values are larger than 150 RU, the binding of these chemicals to rVif is strong. From the 1 ^st^ round screening, 66 compounds were identified to interact with rVif. Subsequently, we hypothesized that if small molecules interrupt the interaction between A3G and Vif, they may have binding abilities to either A3G or Vif. As A3G-NTD is mainly responsible for the interaction with Vif, we tested the binding affinities of these 66 compounds to A3G-sNTD or rVif. From the 2 ^nd^ round screening, 14 compounds in the ZXM group (including ZXM1-1, ZXM1-2, ZXM1-5, ZXM1-12, ZXM1-15, ZXM2-2, ZXM2-3, ZXM2-4, ZXM2-6, ZXM2-9, ZXM2-10, ZXM2-16, ZXM2-18 and ZXM2-20) and 8 compounds (including WL1-3, WL3-7, WL6-3, WL6-4, WL6-6, WL7-2, WL7-3 and WL7-7) in the WL group were found to interact with both A3G-sNTD and rVif, and their *K*_D_ values are listed in the Supplementary Tables S1 and S2). Among these compounds, ZXM1-1 possesses the strongest binding affinities to both A3G-sNTD and rVif, with a *K*_D_^A3G-sNTD, ZXM1-1^ value of 3.5 μM and *K*_D_^rVif, ZXM1-1^ value of 10.1 μM. ZXM2-6 prefers to interact with rVif with a *K*_D_^rVif, ZXM2-6^ value of 8.6±1.9 μM, rather than to bind to A3G-sNTD, with a *K*_D_^A3G-sNTD, ZXM2-6^ value only of 46.7±12.0 μM.

### Compounds restore Vif-suppressed A3G expression

**Figure 2:**
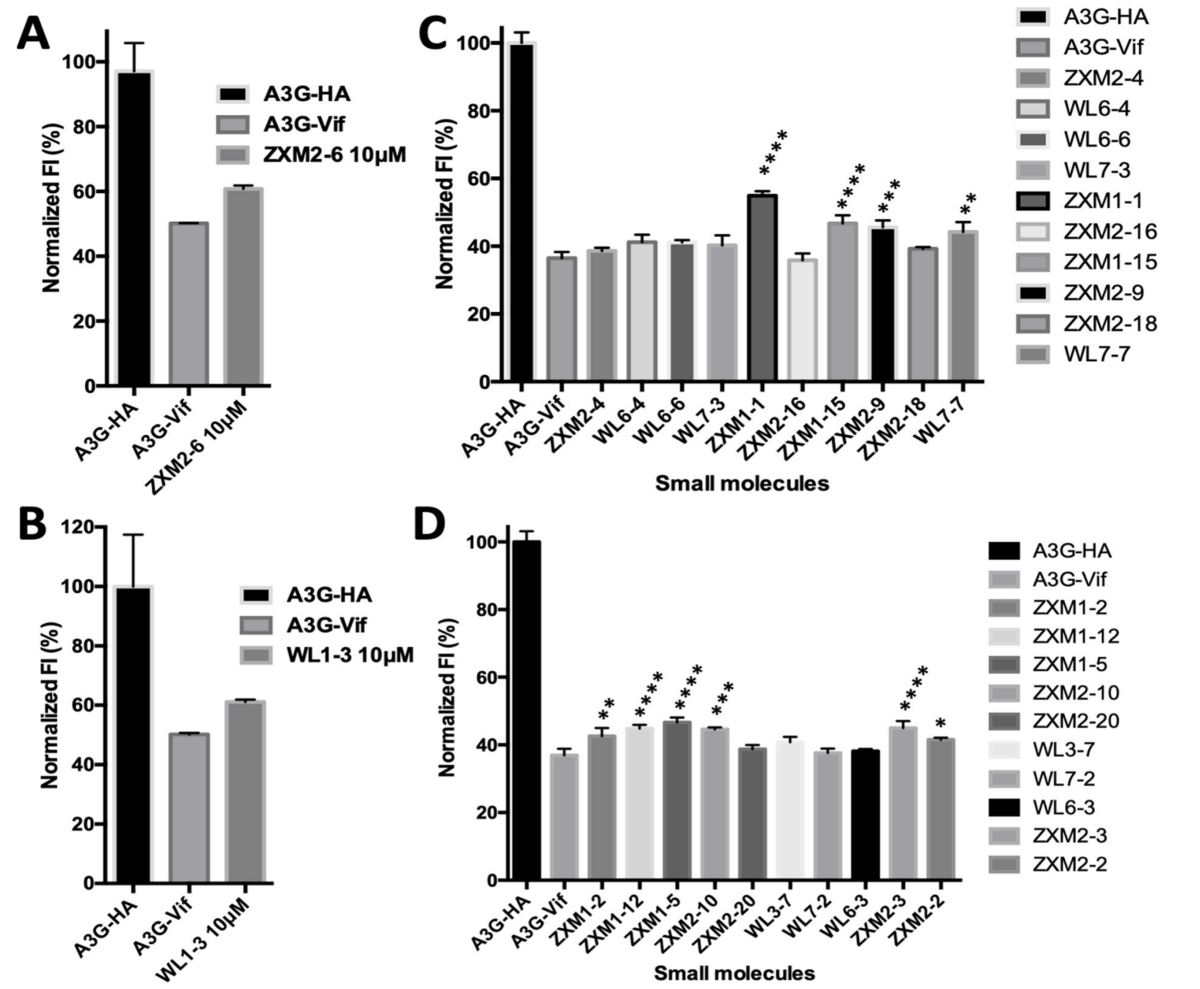
Figure2 Measurements of fluorescent intensity (FI) in 293T cells under the treatment with small molecules FI of cells transfected with A3G-HA and A3G-Vif plasmids were treated with ZXM2-6 (A), WL1-3 (B) and other 20 small molecules (C,D). Vertical axes indicate the normalized FI. The averaged FI of A3G-HA cells is set as 100%; the number of asterisks stands for the statistical difference between the FI of A3G-Vif cells with and without treatment with small molecules. *P<0.05, **P<0.01, ***P<0.001, ****P<0.0001 analyzed by One-way ANOVA, Holm-Sidak’s multiple comparisons method.

**Figure 3:**
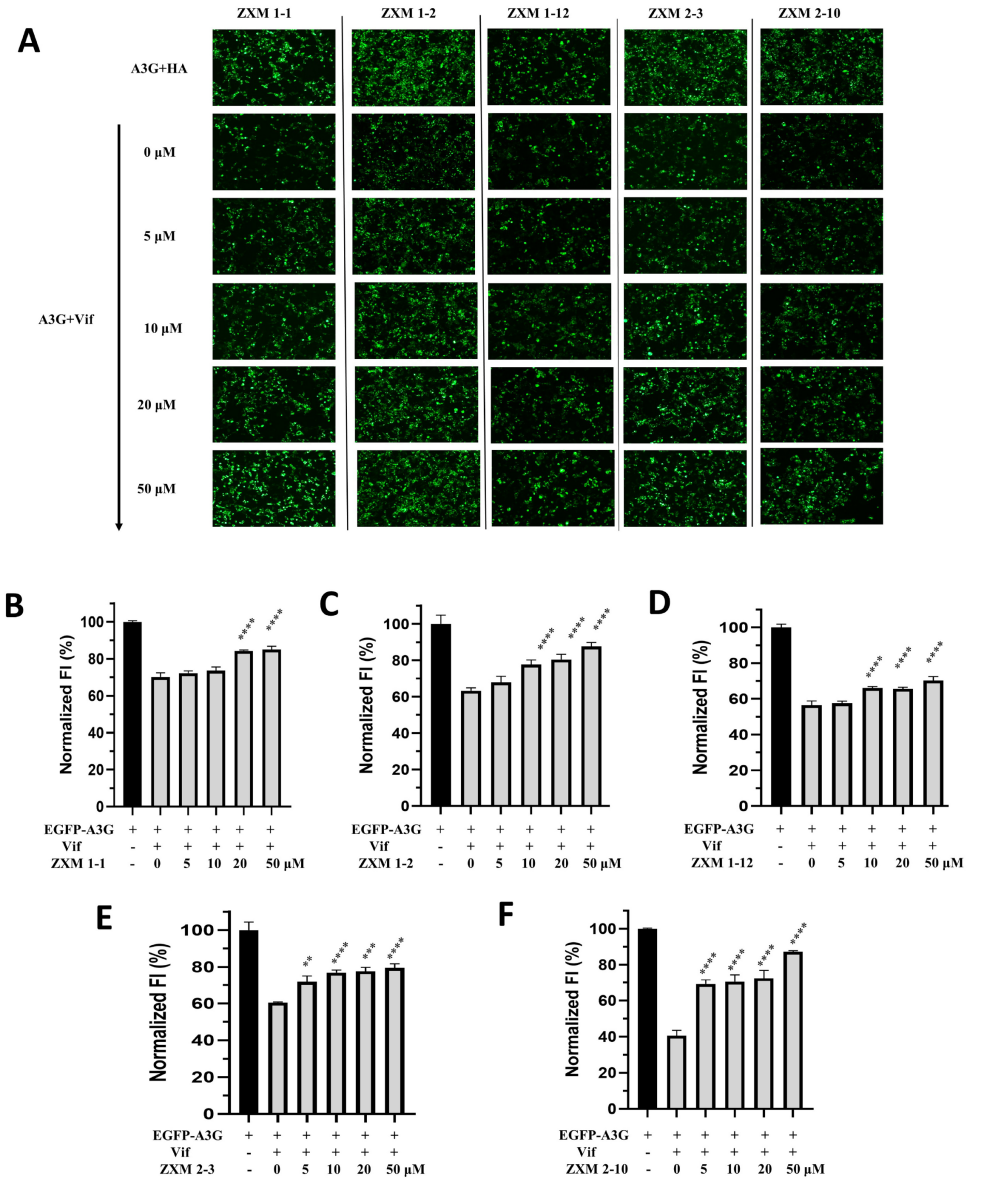
Figure3 EGFP-A3G fluorescence is recovered dose-dependently by small molecules Measurement of FI (A) indicates the dose-dependent recovery of EGFP-A3G fluorescence (down) by five small molecules ZXM1-1 (B), ZXM1-2 (C), ZXM1-12 (D), ZXM2-3 (E) and ZXM2-10 (F), respectively. Vertical axes indicate normalized FI. The averaged FI of A3G-HA cells is set as 100%. Concentrations of small molecules in A3G-Vif cells are 0, 5, 10, 20, 50 μM, respectively. The number of asterisks stands for the statistical difference between the FI of A3G-Vif cells with and without treatment with small molecules. **P<0.01, ***P<0.001, ****P<0.0001 analyzed by One-way ANOVA, Holm-Sidak’s multiple comparisons method.

To testify whether these 22 compounds interrupt the interactions between A3G and Vif in cells, we performed the 1 ^st^ round fluorescence intensity-based drug screening assay ( Figure 2). In this assay, EGFP-fused A3G (EGFP-A3G) was overexpressed in HEK 293T cells together with Vif or the empty vector. EGFP-A3G was supposed to be expressed lower in the presence of Vif in cells due to Vif-triggered polyubiquitination and degradation of A3G. In the presence of effective inhibitors, the fluorescence intensity of the cells was expected to be intuitively recovered. We found that ZXM2-6 was unable to elevate the fluorescence intensity of EGFP-A3G which was co-expressed with Vif, although it displayed strong binding affinity to rVif. Nine compounds, including ZXM1-1, ZXM1-2, ZXM1-5, ZXM1-12, ZXM1-15, ZXM2-3, ZXM2-9, ZXM2-10 and WL7-7, showed the ability to increase fluorescent intensity of GFP-A3G. Then, we carried out the 2 ^nd^ round of the fluorescence intensity-based screening assay, in which different doses of inhibitors were administrated. Five chemicals, including ZXM1-1, ZXM1-2, ZXM1-12, ZXM2-3 and ZXM2-10, were recognized to enhance fluorescent intensity in a dose-dependent manner ( Figure 3), and treatment with these chemicals did not affect the cell growth significantly at concentrations lower than 50 μM ( Figure 3A). Compared with the control group without small molecules, the fluorescence intensity could be enhanced by 10%–50% after treatment with these five small molecules ( Figure 3B–F). Among these five compounds, ZXM1-2 and ZXM2-10 showed more significant effects than other three compounds. The effect of ZXM2-10 was extremely excellent, which could restore about 50% of fluorescent intensity.

**Figure 4:**
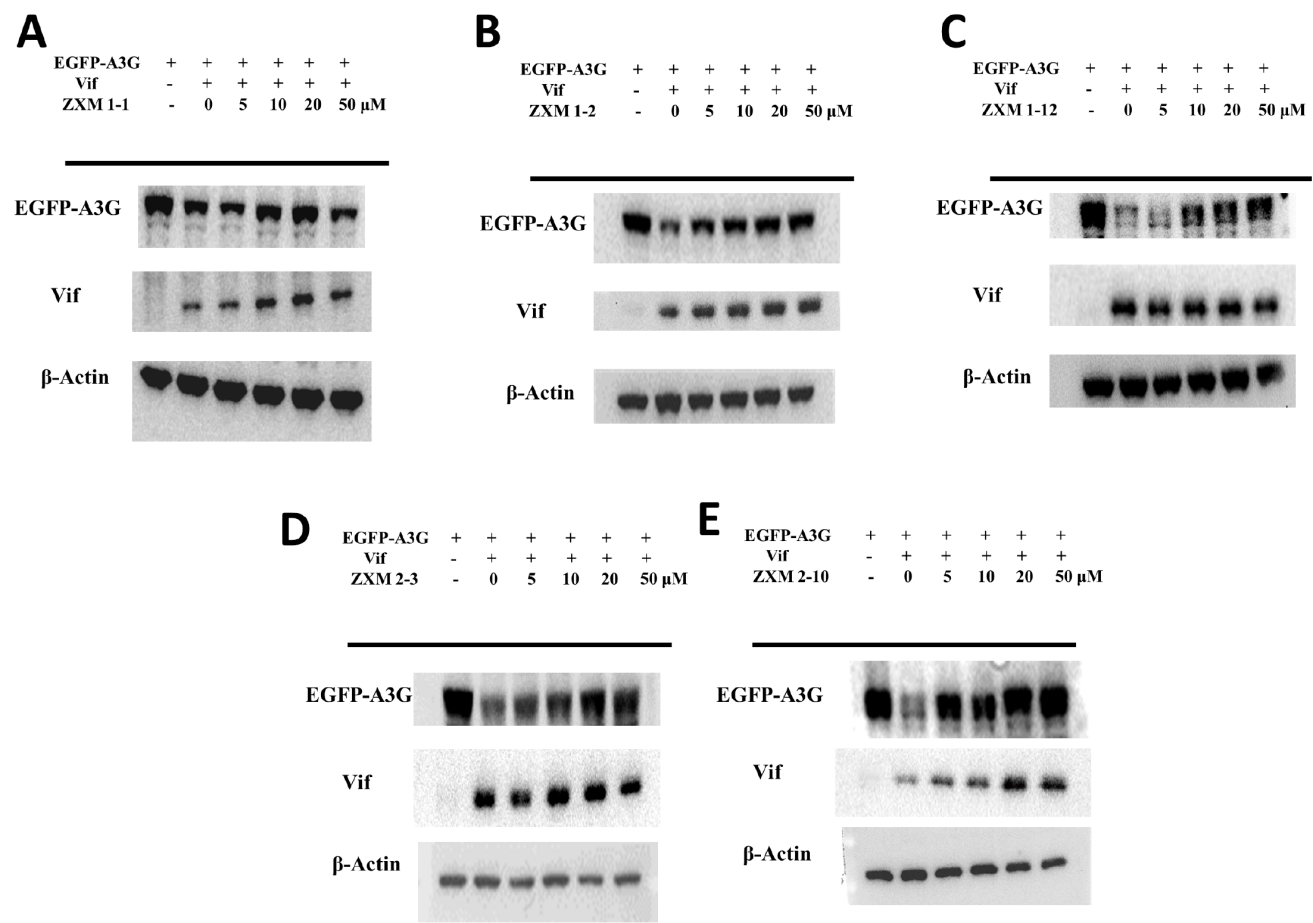
Figure4 EGFP-A3G expression level is increased with increasing concentrations of small molecules Recovery of EGFP-A3G expression levels of under the treatment with the chemicals (A) ZXM1-1, (B) ZXM1-2, (C) ZXM1-12, (D) ZXM2-3 and (E) ZXM2-10 at different concentrations, respectively.

To further explore whether these five small molecules can restore the fluorescence intensity of EGFP-A3G protein by affecting the expression of Vif, and whether the fluorescence recovery is related to the expression of EGFP-A3G, we conducted western blot analysis with the cell lysates. As shown in Figure 4, compared with the A3G+HA group without Vif protein expression, the expression of EGFP-A3G was significantly reduced in cells co-expressed with Vif. At the concentration of 5 μM, ZXM1-2 and ZXM2-10 dramatically enhanced the intensity of EGFP-A3G, indicating that the expression level of EGFP-A3G was increased under the treatment with either ZXM1-2 or ZXM2-10. When the concentration of small molecules was 10 μM, the expression of EGFP-A3G in ZXM1-12 and ZXM2-3 groups were significantly increased. When the concentration of ZXM1-1, ZXM2-3 and ZXM2-10 was increased to 20 μM, the level of A3G expression was close to that of the EGFP-A3G+HA group. When the small molecule concentration reached 50 μM, the EGFP-A3G band was close to that of the A3G+HA group, indicating that the expression level of A3G was remarkably restored. Moreover, small molecules almost did not affect the expression of Vif protein, except that ZXM2-10 slightly increased the expression of Vif in cells through some unknown mechanism. These data are consistent with the results from the fluorescent-intensity based screening assay ( Figure 3), indicating that the recovery of intracellular fluorescence intensity reflects the expression level of EGFP-A3G. Small molecules could effectively restore the expression level of intracellular A3G protein in the presence of Vif protein, which showed stable concentration-dependent characteristics.

### Compounds block the interactions between A3G and VCBC complex

**Figure 5:**
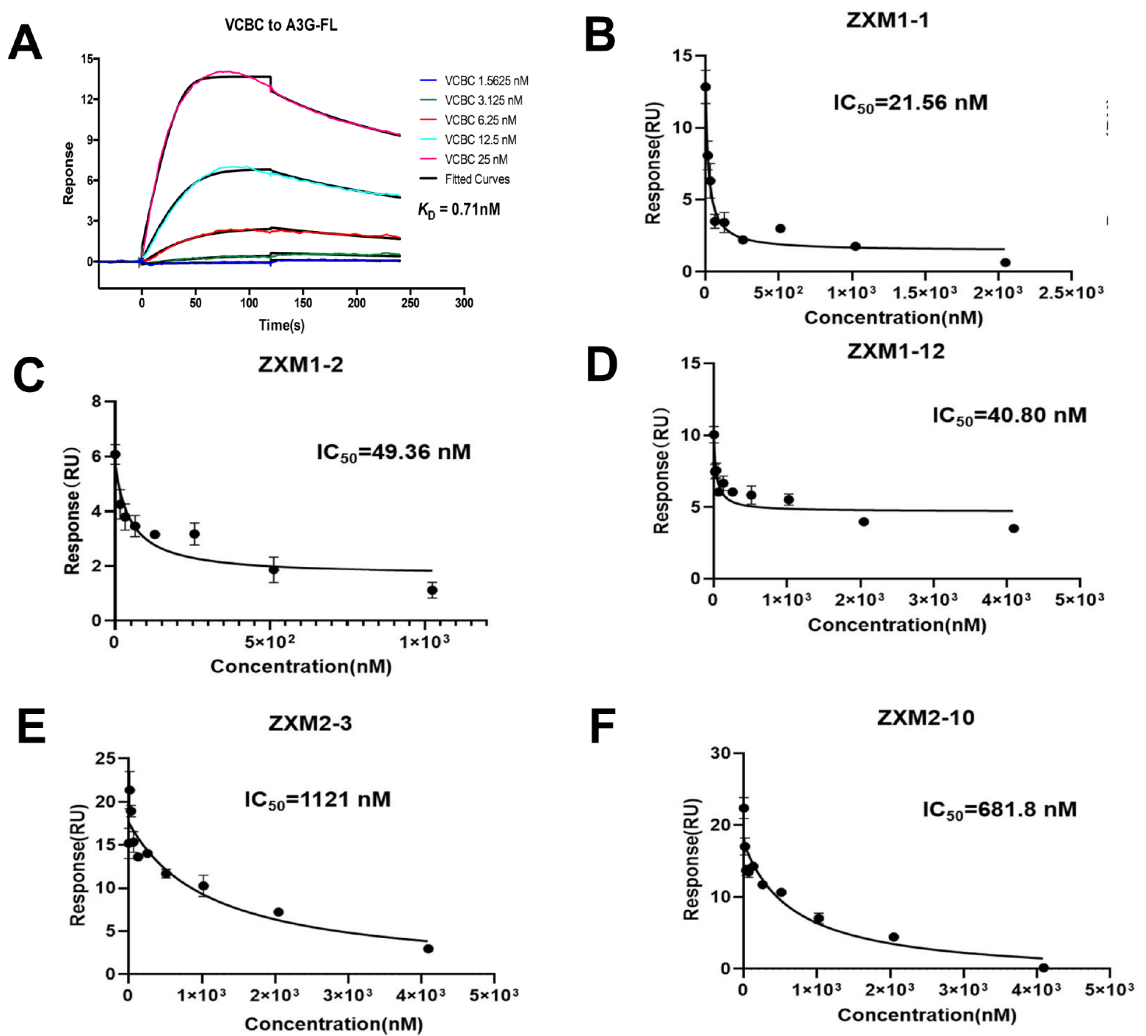
Figure5 Compounds block the interactions between A3G and Vif-CBFβ-ELOB-ELOC (VCBC) complex (A) Kinetics fitting of the binding of A3G-FL with VCBC complex measured by SPR assay. (B–F) The inhibition constants of the chemicals ZXM1-1 (B), ZXM1-2 (C), ZXM1-12 (D), ZXM2-3 (E) and ZXM2-10 (F) against the interaction between A3G-FL and VCBC complex, measured by SPR assay.

To well understand whether the inhibitors disrupt the interaction between full-length A3G (A3G-FL) and Vif, we detected the interaction between A3G-FL and VCBC ( *i*. *e*., Vif-CBFβ-ELOB-ELOC) complex by SPR assay. A3G-FL was purified from Sf9 insect cells, while VCBC complex was directly generated from *E. coli*system, as previously reported [1]. A3G-FL displayed a strong binding affinity to VCBC complex with a *K*_D_^A3G-FL, VCBC^ of 0.71 nM, and an association constant ( *k*_a_) of 5.8×10 ^6^M ^–1^s ^–1^ and a dissociation constant ( *k*_d_) of 0.0041 s ^–1^ ( Figure 5A). Compared with the results shown in Figure 1C, these data implied that A3G-FL could bind to VCBC complex much stronger than to rVif, in line with the high recruitment ability of A3G-FL by VCBC complex in cells.

Subsequently, we performed SPR experiments to test whether these five compounds could inhibit the binding of A3G-FL to VCBC complex. During the measurement of binding affinity *K*_D_ of A3G-FL to VCBC complex ( Figure 5A), A3G-FL on the chip was saturated with only 25 nM VCBC complex. Therefore, in the competitive binding experiments, to ensure that all A3G-FL on the chip bound to VCBC complex, we fixed the concentration of VCBC complex at 32 nM. This could avoid the interference of the response values generated by the combination of small molecules and A3G-FL under unsaturated conditions. The increase of small molecule concentrations led to the decrease of SPR signal, indicating that the small molecule effectively inhibited the binding of A3G to Vif *in vitro*. As shown in Figures 5B–F, these five small molecules significantly reduced the interactions between A3G-FL and VCBC complex in a concentration-dependent manner. Among them, ZXM1-1 displayed the best competitive activity with an IC _50_^ZXM1-1^ value of 21.56 nM. ZXM1-12 (IC _50_^ZXM1-12^=40.8 nM) and ZXM1-2 (IC _50_^ZXM1-2^=49.36 nM) demonstrated much stronger competitive abilities than ZXM2-3 (IC _50_^ZXM2-3^=1.21 μM) and ZXM2-10 (IC _50_^ZXM2-10^=0.68 μM). These results showed that the five chemicals interrupted the interaction between A3G and VCBC complex *in vitro*, while their effects *in vivo* need to be further investigated.

### Compounds inhibit Vif-mediated A3G ubiquitination

**Figure 6:**
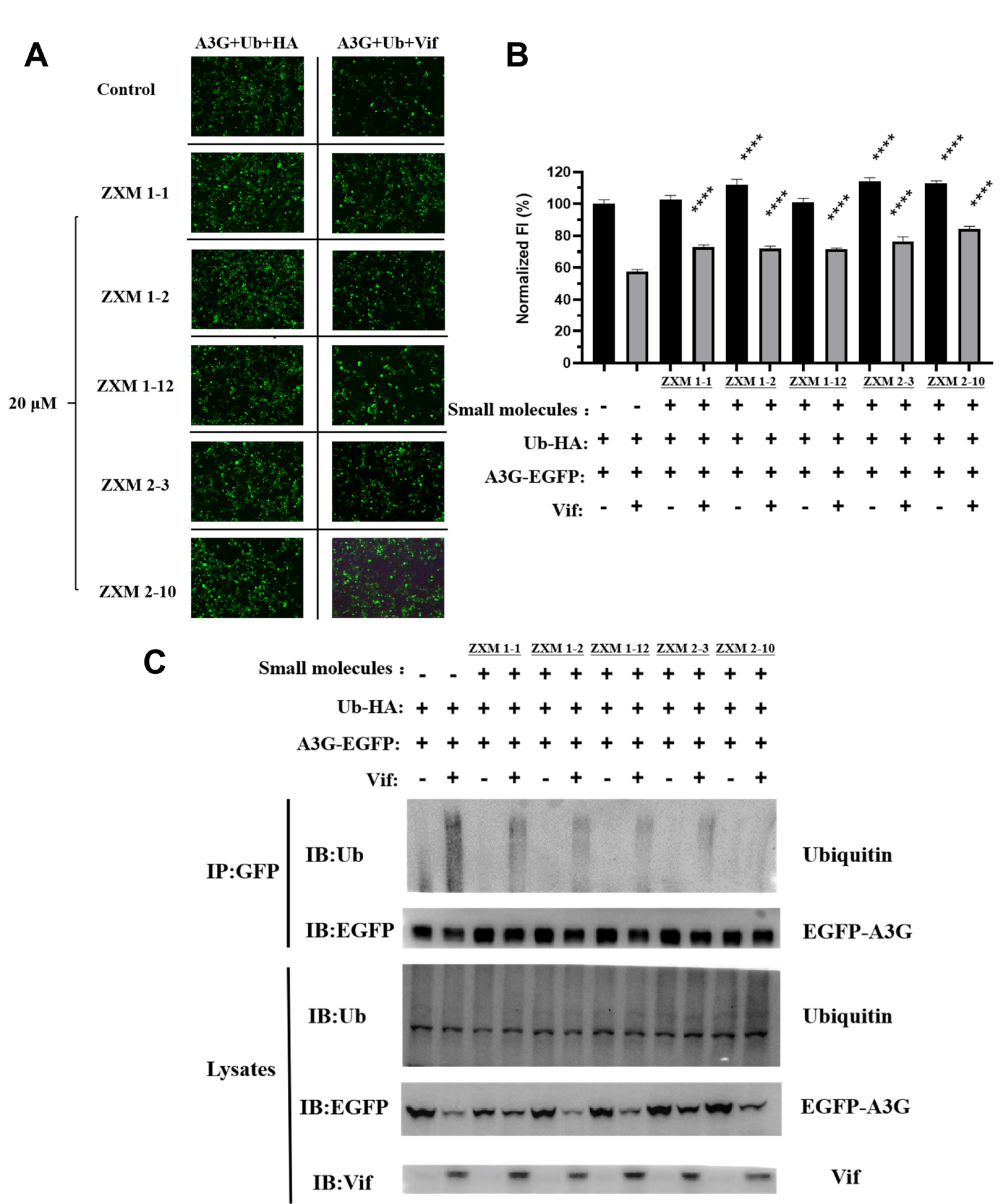
Figure6 Small molecules inhibit Vif-mediated polyubiquitination and degradation of A3G (A,B) Effects of different small molecules on the fluorescence intensity of intracellular EGFP-A3G at a concentration of 20 μM. Vertical axes indicate normalized FI. The average FI of A3G-HA cells without small molecules is set as 100%. The concentration of small molecules in 293T cells is 20 μM. Number of asterisks stands for the statistical difference between the FI of A3G-Ub-Vif cells with and without treatment with small molecules. ****P<0.0001. (C) The small molecules inhibit polyubiquitination of APOBEC3G. 293T cells were transfected with expression vectors for EGFP-fused A3G and HA-tagged ubiquitin, in the presence or absence of co-transfection of the Vif expression vector, and treated with small molecules for 36 h. Cell lysates were immunoprecipitated with anti-GFP antibody and analyzed by western blotting using anti-ubiquitin antibody, anti-EGFP antibody and anti-Vif antibody.

Thus, we further tested whether the up-regulation of A3G expression by small molecules, including ZXM1-1, ZXM1-2, ZXM1-12, ZXM2-10 and ZXM2-3, is related to the Vif-mediated ubiquitin-proteasome degradation process. Since cell growth was not affected after 48 h of treatment when the concentration of small molecules was less than 20 μM ( Supplementary Figure S1), and A3G expression was restored at this concentration ( Figure 3A–F), the transfected cells were then treated with 20 μM of these five chemicals, respectively. As shown in Figure 6A,B, all the five chemicals could restore the fluorescent intensity (FI) of EGFP-A3G from 57% to more than 70%. ZXM2-10 significantly restored FI to more than 80%. EGFP-A3G and ubiquitin were then co-overexpressed with or without Vif, and cells were treated with five chemicals at 20 μM, respectively. EGFP-A3G was immunoprecipitated by anti-GFP antibody, and anti-ubiquitin antibody was used to detect the A3G ubiquitination. The whole cell lysates were used as the expression control ( Figure 6C). The results showed that the global expression of A3G, Vif and ubiquitin (Ub) were all detected in the cell lysates, which proved that the plasmids were successfully transfected and expressed in HEK 293T cells. Compared to the A3G+Ub group without small molecules, Vif promoted the degradation of A3G ubiquitination in the A3G+Ub+Vif group without small molecules, which confirmed that Vif mediated A3G ubiquitination. In contrast, the polyubiquitination smear in the A3G+Ub+Vif group in the presence of different small molecules were observably much weaker than that in the A3G+Ub+Vif group without small molecules, suggesting that these five chemicals could affect the Vif-mediated A3G ubiquitination. This might be consequent on inhibiting the interaction between A3G and VCBC complex. Among the five chemicals, ZXM2-10 displayed the strongest inhibition activity on the Vif-mediated A3G ubiquitination.

## Discussion

In this study, through SPR screening assay and FI based cell biological experiments, we obtained five small molecule inhibitors against the interactions between A3G and Vif, as well as between A3G and VCBC complex. In order to confirm the effect of these small molecules in cells, we investigated their effects on A3G ubiquitination and degradation. Our results showed that the polyubiquitination of A3G were significantly weakened after the addition of these five small molecules, indicating that they could reduce the level of Ub-A3G, and the expression of A3G protein was restored even in the presence of Vif. Ubiquitination experiments enabled us to clearly understand the mechanism of action of these five small molecule inhibitors in cells and provided strong evidence for subsequent studies on the structural basis of their binding to A3G or Vif or VCBC complex. All these five compounds, which contain two different aromatic rings connected by disulfide bond, are inhibitors with relatively novel skeleton against the interactions between A3G and Vif or its VCBC complex.

It is well known that the aromatic disulfides could potentially be covalent inhibitors. Is it possible that these five compounds covalently bind to A3G or Vif in our cases? Generally, from the original SPR data, we can assess whether or not the small compound can covalently bind to its target. If the compound binds to its target tightly or covalently, the dissociation process will be extremely slow, or even cannot be detectable. However, in our case, as seen in the Supplementary Figure S2, we found that all our compounds could dissociate from A3G or Vif, which means they do not covalently bind with A3G or Vif. Thus, these results pave a new way to the discovery of new anti-HIV-1 small molecule drugs.

## Supplementary Data

Supplementary data is available at *Acta Biochimica et Biphysica Sinica* online.
